# Gender Assignment Based on Mannequin Anatomy

**DOI:** 10.1111/tct.70427

**Published:** 2026-04-21

**Authors:** Dowan Kwon, Kirsty Mackay, Christa Brew, Jo Hartland

**Affiliations:** ^1^ Bristol Medical School University of Bristol Bristol UK; ^2^ University Hospitals Bristol and Weston NHS Foundation Trust Bristol UK; ^3^ University Hospitals Birmingham NHS Foundation Trust Birmingham UK

## Abstract

**Background:**

Misgendering transgender people in healthcare contributes to fear of discrimination and stigmatisation, and transgender health inequality. Much of the language used to describe biology is discussed in a cisnormative manner. We explored the language used in a clinical skills laboratory, where pelvic mannequins with external genitalia were used.

**Approach:**

Urethral catheterisation practical sessions were organised for 4th and 5th‐year medical students at a UK undergraduate medical department. Students and facilitators were blinded to the research nature initially. Sessions were directly observed by clinical educators who wrote reflections based on the language used in the classroom. Following the observation, the research details were presented to students. A questionnaire was used to establish students' previous learning experiences of transgender health and the students' awareness of their own language to describe medical mannequins.

**Evaluation:**

Twenty‐one reflections were generated by seven observers across 14 sessions. Fifty‐five students completed the questionnaire. Three main themes were identified from the observer reflections: heavily utilised gendered language, linguistic mirroring between facilitators and students and one‐dimensional task‐focus. Fifty‐two out of fifty‐five (94.5%) of participants responded that they had not previously considered the appropriateness of using pronouns when referring to mannequins.

**Implications:**

We identified the need for systemic changes including relabelling mannequins and procedures from ‘male’ and ‘female’ to using anatomical terms such as ‘penile’ or ‘vulval’ catheterisation. The finding of significant linguistic mirroring shows that better faculty training is necessary. There are also opportunities to introduce transformative learning in clinical skills practice, challenging unhealthy and unhelpful behaviours without compromising skill acquisition.

## 1 Background

In England and Wales, 0.5% of the population (262,000 people) do not identify with the sex registered at birth [1]. Misgendering transgender people in UK healthcare has led to fear of discrimination and stigmatisation [2], negatively impacting transgender people's health outcomes [3], and 41% of transgender people feel healthcare professionals poorly understand their health needs [4]. Discussions about sex and gender remain highly politicised, but within medicine, it is important that we respect how our patients identify and describe themselves. Sex is also not binary [5] but is assigned at birth based on the appearance of external genitalia, using typically male or female characteristics. Gender is socially constructed by how we dress, speak or act, which are all interpreted by those around us in the context of cultural and temporal gender norms. Gender identity describes our own internal relationship with ideas of typical masculinity, femineity and a spectrum of experience in‐between [5]. In our experience, much of the language used to describe biology within medical education is cisnormative, meaning biology and anatomy are often unconsciously discussed in a binary‐gendered manner. This creates a hidden curriculum, which may encourage students to unconsciously exclude transgender people from their clinical reasoning and language, possibly contributing to health inequalities. To investigate this further, we explored the language used in a clinical skills laboratory where intimate examination and procedures were taught using pelvic mannequins, which are commonly referred to, and even marketed as, ‘male’ (with a penis) and ‘female’ (with a vulva) models.

*Gender is socially constructed by how we dress, speak or act, which are all interpreted by those around us in the context of cultural and temporal gender norms*.

## 2 Approach

Voluntary urethral catheterisation clinical skills sessions were advertised by email for 4th‐ and 5th‐year medical students at a UK hospital undergraduate department in Southwest England. Near‐peer clinical educators were recruited as facilitators, demonstrating and supervising catheterisation procedures using mannequins. Each session lasted approximately 40 minutes and involved one facilitator and three to five medical students who were all blind to the study. Two other faculty members observed each session and were instructed to record the language used to refer to the mannequins, including any gendered pronouns. Observers also wrote reflections immediately after each session, documenting personal subjective observations of language use and behaviour. To achieve more naturalistic observation, facilitators were also blinded from the nature of the research and were asked not to interact with the observers.

In total, 14 sessions were run over three days, involving 65 students, four facilitators and seven observers. Twenty‐one observer reflections were generated, and 55 students consented to complete the questionnaire. Only penile catheterisation mannequins labelled ‘male catheterisation mannequin’ were available for teaching. After the session, the research was explained to students and facilitators in separate debriefs, where informed consent was gathered to use observations. This included a micro‐teach session to contextualise the study, illustrating the health inequalities faced by transgender people in the United Kingdom. If consenting, students completed questionnaires exploring experiences with transgender patients and students' perceived use of their own language to describe mannequins during the session.

Content analysis [6] of observer reflections was undertaken by the lead author under the guidance of an experienced qualitative researcher. Synthesised codes of similar nature were tallied to create subthemes and subsequent themes. The analysis was interrogated and challenged by a supervisor prior to final agreement. The team undertaking this work included cis and trans researchers.

Ethical approval for the study was obtained from the University of Bristol Faculty of Health Sciences Research Ethics Committee (Ref. 114344).

## 3 Evaluation

Three main themes were synthesised from observer reflections: gendered language, linguistic mirroring between facilitators and students, and the unrealistic nature of simulated settings (Table 1).

**TABLE 1:** Thematic summary table. Tally of observer reflections shows the number of times the subtheme was described in observer reflections. Total number of observer reflections = 21.

Themes	Subtheme description	Tally of observer reflections	Highlight quotes from observer reflections
Gendered language	Gendered language to describe procedure	8	‘Students spontaneously differentiated between “male” and “female”’ ‘… question about if male or female catheterisation easier’ Facilitator—‘Who has done a catheter before? Was it a man/woman?’
	Gendered language to describe mannequin or the patient	7	‘Students and facilitator referred to the manikin as male using “guy,” “he,” and “Mr”’ Student—‘Is it easier to do a man or a woman’ Facilitator—‘if you have a gentleman …’
Linguistic mirroring	Mirrored language between facilitator and students	8	‘Facilitator repeated language that was used (by the student)’ ‘Students tended to use he/she and facilitator would mirror the language’ ‘The facilitator/students tended to mirror each other in terms of speech’.
One‐dimensional task‐focus	Lack of pronoun use	8	‘… not referring to the person they were catheterising’ ‘Students also never referenced the mannequin here’
	Task‐focussed	6	‘very focused purely on the skill of catheterisation’ ‘Both facilitator and students were very task focused’ ‘They (facilitator and students) were very task focused and rarely referred to the model at all’.

### 3.1 Gendered Language

Gendered language featured heavily during sessions with numerous male descriptors of the penile mannequin, e.g., ‘guy’, ‘Mr’, ‘gentleman’, ‘man’, and ‘he’.

*Gendered language featured heavily during sessions with numerous male descriptors of the penile mannequin, e.g., 'guy', 'Mr', 'gentleman', 'man', and 'he'*.

Procedures were frequently categorised as ‘female’ and ‘male’, with discussions of prior experience of observing or performing catheterisation divided into experiences with female and male patients. Examples of language can be seen in Table 1. Facilitators and students often echoed the male/female mannequin manufacturer description and language used in the medical school‐provided skills booklet (since adapted).

### 3.2 Linguistic Mirroring

Facilitators and students reflected each other's language back during discussions. Notably, when one student specifically used ‘they’ to describe the mannequin, the facilitator mirrored back gender‐neutral language, often using the same vocabulary. Where the facilitator used descriptive language such as ‘the penis’ or ‘the mannequin’, students avoided gender‐specific nouns and pronouns, using these same terms.

*Notably, when one student specifically used ‘they’ to describe the mannequin, the facilitator mirrored back gender‐neutral language, often using the same vocabulary*.

### 3.3 One‐Dimensional Task‐Focus

Whilst catheterising, many students were solely focused on the procedure and rarely addressed the mannequin. One facilitator created a scenario involving a male patient called ‘Jack’, stating ‘he is in urinary retention’. This led students to refer to the mannequin by male pronouns and as ‘Jack’ during the procedure.

#### 3.3.1 Questionnaire Data: Students' Previous Educational Experiences of Transgender Health

**FIGURE 1:**
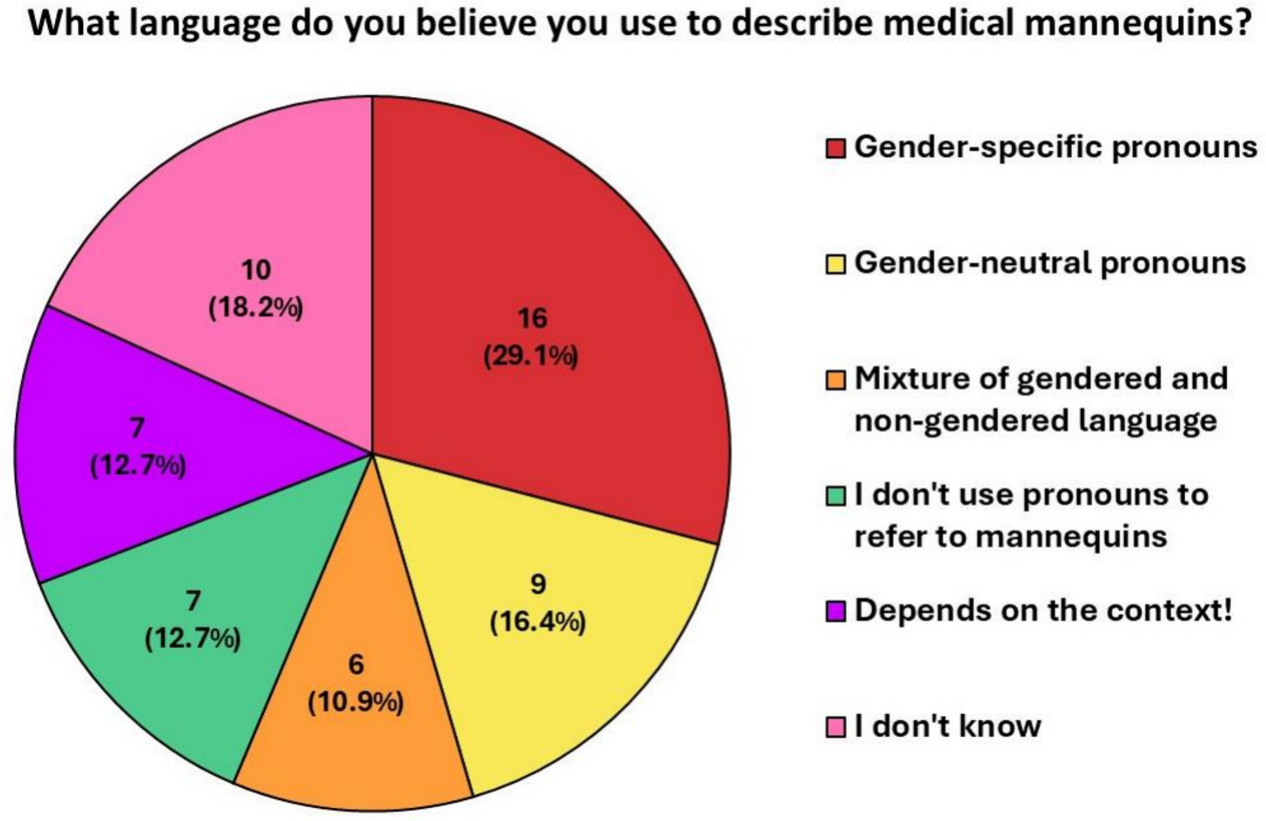
Survey outcome of student response to their use of language to describe medical mannequins (*n* = 55). Gender‐specific pronouns (16, 29.1%), gender‐neutral pronouns (9, 16.4%), mixture of gendered and nongendered language (6, 10.9%), I do not use pronouns to refer to mannequins (7, 12.7%), Depends on the context! (7, 12.7%) and I don't know (10, 18.2%).

**FIGURE 2:**
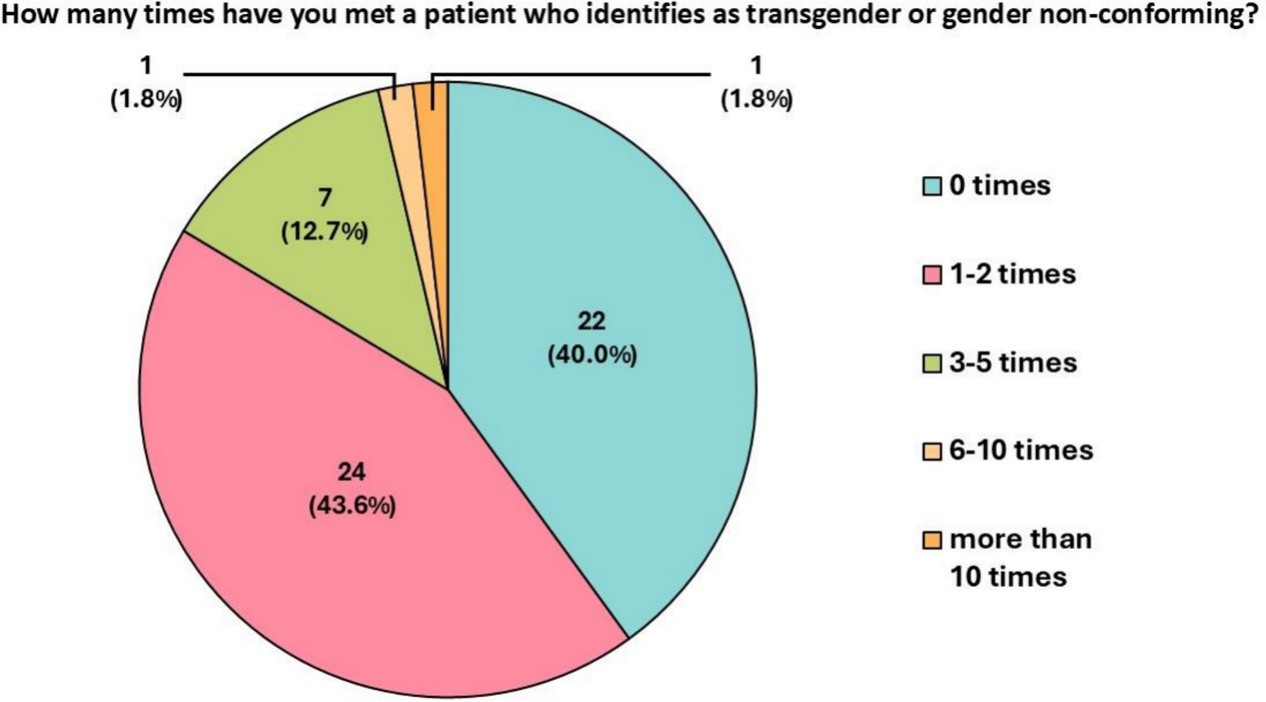
Survey outcome of student response to the frequency of meeting transgender or gender nonconforming patients (*n* = 55). Zero times (22, 40.0%), 1–2 times (24, 43.6%), 3–5 times (7, 12.7%), 6–10 times (1, 1.8%) and more than 10 times (1, 1.8%).

Fifty‐two out of fifty‐five (94.5%) student participants had not previously considered the appropriateness of using pronouns to describe mannequins. Questionnaires revealed a wide variety of student beliefs regarding their own default language (Figure 1). Those who believed they would use gender‐specific pronouns to address medical mannequins frequently cited unconscious bias as the reason. Twnety‐two out of fifty‐five (40.0%) responded that they had either never come across a patient who identifies as transgender or gender nonconforming, with 24/55 (43.6%) having done so 1–2 times (Figure 2).

## 4 Implications

There are numerous implications for clinical educators to consider based on our findings.

### 4.1 Need for Systemic Changes

During our curriculum teaching on transgender health, students are taught not to associate genitals with gender identity, and yet there appears to be tension with the approach used in clinical skills teaching. In the context of substantial transgender health inequality, we should ensure that inclusive education is delivered consistently across our curricula.

It is more accurate, safer and inclusive of trans and intersex biology to describe anatomy in clinical skills teaching: for instance, a catheter being more difficult due to the presence of a prostate, not whether a patient is male or female. Mannequins should be labelled based on anatomy, rather than their non‐existent gender, and medical school's skills logbook and assessments should also reflect this anatomical focus.

*It is more accurate, safer and inclusive of trans and intersex biology to describe anatomy in clinical skills teaching: for instance, a catheter being more difficult due to the presence of a prostate, not whether a patient is male or female*.

Our questionnaire showed that students believe they have rarely met transgender or gender nonconforming patients (Figure 2). The Office of National Statistics [1] shows that eight out of the ten local authorities with the largest proportions of people identifying as transgender in England and Wales are in London. This urbanisation and variability of transgender populations further highlight the importance of medical education that is inclusive of transgender people across all medical education institutions, ensuring that such teaching is not limited by access to local demographics.

### 4.2 Adequate Faculty Training Required

Linguistic mirroring was a crucial finding. Despite students' perceptions of their own language use (Figure 1), gendered language was used heavily throughout the observed sessions, and students specifically imitated the gendered language facilitators initiated during teaching. Adequate faculty training benefits students and their learning [7, 8, 9], and we cannot expect students to overcome this bias if we do not counter the cisnormative training most qualified clinical educators are likely to have experienced in their own medical schools.

### 4.3 Transformative Learning

The micro‐teach on transgender health following the practical session introduced transformational learning in practice [10]. It allowed educators to expand a clinical skills session and into a reflection on bias in clinical practice. Such teaching [our transformational learning in practice] challenges students to think more inclusively, create solutions in a learning community and appreciate how health inequalities faced by minority populations can be perpetuated by our curriculums. Even a course designed with the best intentions might have unintended consequences for students [11], and as educators, we can inadvertently pass on cultural or moral values that contradict explicit learning objectives [12].

*Such teaching [our transformational learning in practice] challenges students to think more inclusively, create solutions in a learning community and appreciate how health inequalities faced by minority populations can be perpetuated by our curriculums*.

We are not alone in suggesting that when themes of equity, diversity, and inclusion are explicitly incorporated into simulation‐based education; it can increase student self‐awareness, communication, insight and knowledge [13]. We believe high‐ and low‐fidelity simulation using mannequins can expose such hidden curriculum and reduce the possibility of students taking on exclusionary practice if we take the time to acknowledge our own problematic language and behaviour. Such transformational learning occurs without sacrificing other aspects of teaching; in this case, students still practised their core clinical skills. However, through the micro‐teach, they also encountered thought‐provoking notions on how we assign gender to objects based on our own learned behaviour, and how this might harm our transgender patients.

### 4.4 Limitations

Although the intention was for all participants to be blinded, some facilitators understandably showed unease at peer observation. Two of the clinical educators were informed that their language around gender will be observed in advance of facilitating the sessions, which may have affected the language used.

## 5 Conclusions

This study provides an insight into how gender may be unconsciously assigned based on external genitalia in an undergraduate medical education setting. A simulated clinical skills session can involve a potentially harmful hidden curriculum, reinforcing strong associations between external genitalia and gender identity. This association could potentially contribute to a vicious cycle of healthcare exclusion and anxiety for transgender people who are unintentionally misgendered during consultations, experience dysphoria from gendered language or are even outed without their consent. We argue that the critical information in successfully catheterising someone has little to do with the person's gender, rather whether there are anatomical variations that affect the procedure. We also believe that there are opportunities in undergraduate settings to introduce transformative learning in clinical skills practice, challenging unconscious bias without compromising skill acquisition.

## Author Contributions

**Dowan Kwon:** conceptualization, investigation, writing – original draft, writing – review and editing, visualization, methodology, project administration, formal analysis, data curation, resources. **Kirsty Mackay:** conceptualization, data curation, investigation, resources, writing – review and editing. **Christa Brew:** data curation, investigation, resources, writing – review and editing. **Jo Hartland:** conceptualization, methodology, supervision, writing – review and editing.

## Funding

The authors received no financial support for the research, authorship, and/or publication of this article.

## Conflicts of Interest

The authors declare no conflicts of interest.

## Data Availability

The data that support the findings of this study are available from the corresponding author upon reasonable request.
